# Pre-exposure prophylaxis awareness, interest, and modality preferences to prevent HIV among cisgender women in the Dominican Republic and the United States

**DOI:** 10.3389/fgwh.2026.1817411

**Published:** 2026-10-21

**Authors:** Deanna Kerrigan, Wendy Davis, Allison O’Rourke, Noya Galai, Andrea Mantsios, Rachel K. Scott, Patricia Moriarty, Aimee A. Metzner, Alan Oglesby, Tahilin Sanchez Karver, Tamara Taggart, Clare Barrington, Breana J. Uhrig Castonguay, Kimi Yonamine, Hoisex Gomez, Martha Perez, Yeycy Donastorg

**Affiliations:** 1 School of Public Health, Rutgers, The State University of New Jersey, Piscataway, Washington, DC, United States; 2 Bloomberg School of Public Health, Johns Hopkins University, Baltimore, MD, United States; 3 Kephi Public Health, New York, NY, United States; 4 MedStar Health, Washington, DC, United States; 5 ViiV Healthcare, Durham, NC, United States; 6 Gillings School of Global Public Health, University of North Carolina, Chapel Hill, NC, United States; 7 Instituto Dermatológico y Cirugía de Piel, Santo Domingo, Dominican Republic

**Keywords:** HIV, preferences, PrEP, social determinants, side effects, women

## Abstract

**Introduction:**

Significant inequities in HIV incidence and prevalence exist among cisgender women (hereafter women), including a disproportionate burden of the epidemic among Black women in the United States and female sex workers globally. Women have limited awareness of and access to pre–exposure prophylaxis (PrEP) for HIV pharmacoprevention.

**Methods:**

We explored PrEP awareness, interest, and modality preferences [daily oral vs. long–acting injectable (LAI) PrEP] in Santo Domingo, Dominican Republic (DR) and Washington, DC (DC) in the United States through in–depth interviews (IDIs) with women (*N* = 40) and healthcare providers (HCPs; *N* = 20) in 2021–2022 and surveys of women (*N* = 602) in 2022–2023. Thematic analysis andmultivariate regression were utilized.

**Results:**

Across settings, women were aged ≥18 years and HIV–negative; most were Black or multiracial, single, in their 30s, with >1 child. In IDIs, women characterized PrEP as an empowerment tool and welcomed modality options; HCPs across sites relayed a lack of comfort and preparation to address the unique needs of women and discuss LAI PrEP. In surveys, women reported low PrEP awareness and use: 29% (DC) and 6% (DR) had heard of daily oral PrEP, <6% had heard of LAI PrEP across sites, and 3% (DC) and 1% (DR) had ever used PrEP. Strong interest in taking PrEP varied (DC, 5%; DR, 80%). Modality preferences were similar, with most preferring LAI vs. daily oral PrEP (DC, 73%; DR, 71%). Across sites, preference for LAI PrEP was inversely associated with concern for injection pain/tenderness. In DC, LAI preference was associated with a higher score on a PrEP optimization/ considerations scale. In the DR, LAI preference was associated with injectable medication/contraception experience and alcohol use and inversely associated with PrEP stigma and partner violence.

**Discussion:**

Diverse groups of women have shared and unique drivers of PrEP modality preferences which should be considered when developing and tailoring multi–level interventions to promote PrEP awareness and uptake among women. These interventions should address social determinants and side effects and include HCP training and tools that promote shared decision–making.

## Introduction

In 2023, cisgender women (hereafter referred to as women) and girls comprised 44% of new HIV acquisitions globally (1) and 19% of new HIV acquisitions in the United States (US) (2). Specific subgroups of women, including Black women in the US and female sex workers globally, have a disproportionately heightened risk of HIV acquisition within and across settings (3, 4). Black females aged ≥13 years in the US have an HIV diagnosis rate of 19.6/100,000 compared with 5.1/100,000 among all females aged ≥13 years (3). Female sex workers globally have a 30-fold higher risk of acquiring HIV than the general female population (4).

Daily oral pre-exposure prophylaxis (PrEP) to prevent HIV acquisition was first approved by the US Food and Drug Administration (FDA) in 2012 (5). In both the US and the Dominican Republic (DR), oral PrEP is available for use (5, 6); however, significant inequities in PrEP use persist. Only 8% of daily oral PrEP users in the US are women (2); moreover, Black women comprise just 26% of female PrEP users, compared with 48% of White female PrEP users, in the US (7). In contrast, global PrEP use is higher among women compared with men (8). A study of countries reporting sex/gender data found that among 123,939 PrEP users, 62% were women (9). Despite this, PrEP use varies widely across countries (9) and within subpopulations of women, such as female sex workers (10), and data on these groups remain limited. In the DR, the cumulative number of people initiating oral PrEP as of November 2024 was 4,746 (6); however, unlike in the US (2, 7), data on PrEP use by sex or gender and subpopulation are not reported.

A range of barriers from individual to social-structural hinder oral PrEP adoption and adherence among women (11–15). Adherence to oral PrEP among women remains suboptimal (11–13), often due to limited PrEP knowledge (13, 14), concerns about side effects (13, 14), and low perceived HIV risk (13). These challenges are further compounded by social-structural factors, including HIV- and PrEP-related stigma (11–14), inequitable gender norms (13, 14), and medical provider prescribing bias (13, 15). In prior research some women have reported that alternative PrEP formulations such as long-acting injectable (LAI) PrEP may reduce stigma and increase privacy and autonomy (10, 16). The FDA approved every-2-month LAI PrEP in 2021 (17, 18), but it is not yet available in the DR (6). In the US, recent data indicate that approximately 17% of those using LAI PrEP are women (19); although higher than that of daily oral PrEP, this rate is still low given the HIV burden among US women, particularly Black women. In the DR, LAI PrEP has yet to be introduced (6); however, our prior work indicated strong interest among female sex workers (16).

Despite the promise of PrEP to reduce HIV inequities among women, the preferences of women for daily oral vs. LAI PrEP have not been well explored, especially outside clinical trial settings. Moreover, limited research exists on understanding the unique barriers and preferences of Black women in the US and female sex workers globally. Additionally, although LAI PrEP offers a promising alternative to daily oral PrEP, little is known about real-world interests, perceptions, and factors influencing its uptake among women. This mixed-methods study helps fill these gaps by exploring modality perceptions and preferences among women across 2 distinct settings—Washington, DC (DC), and the DR—providing an understanding of the drivers of women's PrEP modality preferences and factors that may serve as facilitators or barriers to PrEP uptake across settings and subgroups of women. By examining two samples of women from differing backgrounds in parallel the study provides a unique opportunity to compare and contrast the PrEP related dynamics pertinent to tailoring future interventions. Additionally, through incorporating both patient and provider perspectives, this study highlights key individual and social-structural determinants that influence PrEP interest and adoption.

## Materials and methods

### Study design, setting, and samples

This was an observational, mixed-methods study. We first conducted one-time, semi-structured, in-depth interviews (IDIs) with women with indications for PrEP and their healthcare providers (HCPs) to characterize perspectives and experiences regarding daily oral and LAI PrEP. We leveraged qualitative findings from the IDIs to develop a structured cross-sectional survey administered to women to assess awareness and interest in PrEP and factors associated with preferences for daily oral or LAl PrEP. Both the IDIs and cross-sectional survey were conducted in DC in the US and in Santo Domingo in the DR.

For both stages of the study, we worked with existing partners in DC (MedStar Health) and the DR [Instituto Dermatológico y Cirugía de Piel (IDCP)]. MedStar Health is a not-for-profit health care organization with over 120 facilities throughout DC, Maryland and Virginia in the US. The reproductive health/family planning clinic from which we recruited generally serves low-income women in the greater DC area. IDCP is a not-for-profit health care organization with over 9 clinical care facilities throughout key regions and cities in the DR. They serve as the research hub for numerous HIV related studies and have a long partnership with community groups serving female sex workers, and recruitment occurred through these networks.

Across both settings in the qualitative study, we aimed to recruit approximately 40 women with indications for PrEP and 20 HIV and reproductive health HCPs. Women were purposively sampled. Using a stratified approach we sought a sample of women of whom approximately half reported never having used any form of PrEP previously and the other half reported being prior or currently self-identified PrEP users. Healthcare providers were involved in PrEP care at the study sites but were not necessarily the women's individual HCPs. These providers (10 per site) were nominated by the site PIs or study staff at the clinics in DC and the DR. We monitored saturation of themes through the IDI data collection process for women and providers by generating narrative summaries for each interviews and through team discussions.

The cross-sectional survey included women with indications for PrEP. In DC, participants were sampled from the reproductive health/family planning clinic at MedStar Health by MedStar clinic staff and student research assistants. In the DR, peer navigators working with IDCP and a local community-based organization serving female sex workers recruited participants by leveraging their social networks and relationships in the community.

The following were inclusion criteria for IDIs: cisgender women aged ≥18 years who had an HIV-negative or unknown status; had self-identified factors for HIV vulnerability [i.e., self-reported female sex worker, received diagnosis of sexually transmitted infection (STI) in past 6 months, received treatment for STI in past 6 months, or self-reported multiple sex partners in past 6 months]; were receiving care at a participating clinic; and were either non-PrEP users, currently on PrEP, or had prior PrEP experience. For the survey, inclusion criteria were cisgender women aged ≥18 years who had an HIV-negative status and were receiving care at a participating clinic. Inclusion criteria for HCPs were physicians, physician assistants, nurses, nurse practitioners, and pharmacists involved in HIV or reproductive healthcare.

### Ethics approval and consent to participate

The institutional review boards of the George Washington University in Washington, DC, USA and the IDCP and the Consejo Nacional de Bioética en Salud in Santo Domingo, DR reviewed and approved the study in accordance with the ethical code of the Declaration of Helsinki. Study team members trained in ethics and qualitative and survey research obtained oral consent from participants before initiating the IDIs in both settings and the survey in the DR, as it was interviewer administered. For the survey in DC, as it was self-administered via tablet, potential participants “clicked agree” after reading the consent form.

### Data collection

#### In-depth interview procedures and domains

In-depth interviews were conducted via Zoom or recorded phone call in DC and in-person in the DR by trained qualitative researchers between 11/19/2021 and 02/09/2022 (before and shortly after LAI PrEP was approved in the US in December 2021). There were no other procedural differences in terms of IDI data collection across sites. All IDIs were transcribed verbatim. Separate semi-structured interview guides were used for IDIs with women and HCPs. The guides were developed based on key themes raised by participants in prior formative research (20), including work conducted among female sex workers in the DR and Tanzania (10, 16, 21). The interview guide for women elicited a general description of the participant's experience with HIV prevention strategies over time to provide context to IDI findings. Perceptions of vulnerability to HIV and how perceived vulnerability shaped views of PrEP modalities were explored. Relationship- and gender-related dynamics were also explored as contextual factors shaping PrEP attitudes and decision-making. Participants were asked how LAI PrEP would fit into their lives and in relation to domains such as convenience, confidentiality, daily stressors, and stigma compared with daily oral PrEP and other HIV prevention options. Interviewers explored how participants communicate with their HCPs to make decisions regarding their HIV prevention methods, as well as their perceptions of daily oral and LAI PrEP. The interview guide for HCPs assessed perspectives on daily oral and LAI PrEP, potential advantages and disadvantages of each modality, barriers and facilitators to delivery, and communication/shared decision-making.

### Survey procedures

Based on findings from the qualitative research and literature, survey questions sought to examine specific factors that may drive PrEP preferences. Surveys in DC were conducted on tablets and self-administered. In the DR, interviewers assisted with survey completion using tablets. Participants were surveyed between 01/27/2023 and 07/06/2023 in DC and between 11/15/2022 and 01/26/2023 in the DR.

The main survey domains assessed across both geographic settings included sociodemographic characteristics [e.g., age, race/ethnicity (self-reported in DC and assigned in the DR), education, number of children, etc.], health-related and sexual behavior, perceived HIV risk, and PrEP experience and preferences. Additional independent variables related to sex work as an occupation were included in the DR survey. These covariates were selected based both on the literature as well as the current and our prior qualitative research in each setting. We also employed several latent constructs in the form of scales. We utilized an 8-item PrEP optimization scale related to key considerations when making PrEP decisions (e.g., convenience, side effects, stigma). This measure was adapted from a similar measure of factors driving LAI antiretroviral therapy decisions, which was developed for a National Institutes of Health–supported LAI antiretroviral therapy study (22, 23). Using reliability testing we established the Cronbach's alpha for the current study which was 0.90 in DC and 0.64 in the DR. Higher scores on the PrEP optimization scale indicate that participants consider more of the items on the scale to be important in their decision-making regarding their choice of PrEP; lower scores indicate that fewer items were salient in their decision-making and preferences. After dropping an item through reliability analysis, the range for the final 7-item scale of PrEP optimization was 7–28, with each question having 4-point Likert scale response options. The survey also included an aggregate 7-item PrEP stigma scale which was adapted from an existing HIV stigma scale (24). Using reliability testing we found a Cronbach's alpha of 0.92 in DC and 0.74 in the DR for the PrEP stigma scale. The range for the 7-item PrEP stigma scale was 7–28, with each question having 4-point Likert scale response options. Higher scores on the PrEP stigma scale indicate higher levels of internalized PrEP stigma, whereas lower scores indicate lower levels of internalized PrEP stigma. Mean score on the PrEP optimization scale was 22.1 in DC and 17.3 in the DR. Mean PrEP stigma scale scores were 13.5 and 10.6 in DC and the DR, respectively.

Given the distinct socio-cultural and political contexts of both settings, additional questions relevant to each setting (e.g., types of health insurance) or population group (e.g., types of partners for female sex workers in the DR) were included. The DR survey also included a sex work stigma scale used in previous studies conducted by the DR research team (25), which included domains related to perceived stigma (Cronbach's alpha, 0.87), internalized stigma (Cronbach's alpha, 0.86), and enacted stigma (Cronbach's alpha, 0.89).

Questions about whether before the interview participants had heard of daily oral PrEP and LAI PrEP were set up with the following language: “As you may already know, the CDC [Centers for Disease Control and Prevention] now recommends that all sexually active adults should be informed about the availability of PrEP to protect against acquiring HIV. PrEP is medicine used as part of preventive care before exposure to HIV and is currently available as either a pill taken by mouth every day or an injection into the muscle of the butt given every 2 months at the clinic”. In this survey, these options are called oral PrEP and LAI PrEP. The main outcome variable of interest for the survey was preferred PrEP modality, which was based on the following question: “If you were given a choice between an injectable PrEP medication given every 2 months OR taking PrEP pills every day, would you have (1) a strong preference for an injectable PrEP medication given every 2 months, (2) some preference for an injectable PrEP medication given every 2 months, (3) some preference for taking PrEP pills every day, or (4) a strong preference for taking PrEP pills every day?” The outcome was defined as a binary variable by combining responses 1 and 2 vs. 3 and 4.

### Data analysis

#### In-depth interview analysis

In-depth interviews were analyzed in several phases to facilitate an iterative, abductive process within and across sites. During data collection, interviewers wrote detailed field notes and a narrative summary using a cross-site template immediately after the interview. The study team engaged in weekly debriefing sessions during fieldwork to compare findings, identify emergent themes and areas for further exploration, and ensure key domains needed for the quantitative survey (e.g., the potential importance of different attributes) were being covered. Drawing on salient themes in the field notes and narrative summaries, separate codebooks were developed for women and HCPs. Codes were applied to the text using ATLAS.ti software (26). Memos were written throughout the coding process to document emerging themes and connections (27). Codebooks were modified as needed during the coding process. Once transcripts were coded, key patterns and themes emerging from the code output were synthesized, and salient themes were compared between study sites and across population groups.

### Survey data analysis

Descriptive statistical analyses were performed to assess the distribution of all variables, identify errors and outliers, and combine sparse categories. We categorized continuous variables with skewed distributions based on percentiles of the sample (e.g., tertiles, median). After assessing reliability, summary variables were created for multi-item measures including the PrEP optimization and stigma scales. These summary variables were created by generating a composite score for each participant based on their responses to all scale items.

Multivariate logistic regression was used to evaluate key drivers of PrEP preferences, controlling for relevant sociodemographic variables within and across geographic settings. Analysis was performed separately by geographic site to account for inherent differences between the 2 populations and societal contexts. Bivariate associations of covariates of interest were explored in relation to the main outcome of PrEP preference using unadjusted logistic regression with estimated odds ratios (ORs), 95% CIs, and *P* values. All variables that had an association with the outcome with a *P* value ≤0.2 were entered into an initial multivariable logistic regression model, with the exception of variables such as “fear of needles,” which were too proximal to the outcome of interest. To obtain final parsimonious models that included the most important covariates associated with LAI PrEP preference, stepwise procedures were applied. Final results are given with an adjusted OR (aOR), 95% CI, and *P* value. We did not approach the analysis with specific hypotheses to test but rather a working list of factors, based on the literature and our qualitative work, to explore through the logistic regression modeling.

## Results

### Qualitative findings

Characteristics of the qualitative sample (women, *N* = 40 [DC, *n* = 20; DR, *n* = 20]; HCPs, *N* = 20) are shown in Table 1. Women across both sites had the same mean age of 33 years (range, 20–66 [DC] and 20–58 [DR]). Half (50%) were single in DC and 70% were single in the DR. Women from DC had 1.6 children on average, compared with 2.8 children in the DR. Most (80%) DC women were high school graduates, compared with 50% of DR women. In DC, 95% of women were Black; in the DR, 100% of women were Latina and multiracial. As noted, participants were purposively sampled based on PrEP use. In DC, 30% of women reported former or current PrEP use, compared with 50% in the DR. Healthcare providers were similar across sites regarding demographics; most were women and either physicians or nurses.

**Table 1:** Characteristics of qualitative sample.

Characteristic	Women (*N* = 40)	
	DC (*n* = 20)	DR (*n* = 20)
Age, mean (range), years	33 (20–66)	33 (20–58)
Race^a^, *n* (%)		
Black	19 (95)	0 (0)
Multiple races	0 (0)	20 (100)
Declined to respond	1 (5)	0 (0)
Ethnicity^a^, *n* (%)		
Hispanic/Latina	0 (0)	20 (100)
Highest education		
Primary school	4 (20)	10 (50)
High school	13 (65)	8 (40)
College	3 (15)	2 (10)
Marital status		
Single	10 (50)	14 (70)
Married/Partner	10 (50)	6 (30)
Number of children, mean (range)	1.6 (0–8)	2.8 (0–6)
	Healthcare providers (*N* = 20)	
	DC (*n* = 10)	DR (*n* = 10)
Gender, *n* (%)		
Male	3 (30)	4 (40)
Female	7 (70)	6 (60)
Provider type, *n* (%)		
Health administrator	0 (0)	1 (10)
Nurse	2 (20)	–
Nurse practitioner	3 (30)	–
Psychologist	0 (0)	2 (20)
Physician	5 (50)	7 (70)

DC, Washington, District of Columbia; DR, Dominican Republic.

a Race and ethnicity were self-reported in DC; whereas they were assigned in the DR.

### Low PrEP awareness and lack of women–specific messaging

Among the women participating in qualitative interviews without prior PrEP experience in DC, there was low PrEP awareness in general. Participants in DC who had heard of PrEP generally saw it as a medication intended for men who have sex with men or transgender women. This perception was based on exposure to commercials, health department materials, and members of their social network.

“From what I know, PrEP is like a drug that you can take to prevent your partner from transmitting HIV is what I think it is. I think that was explained to me… I have friends [who] are gay, so they take it”—DC woman, age 38

Healthcare providers in DC echoed the lack of knowledge of PrEP among their female patients and cited non-representation in marketing. Inadequate focused efforts and messaging to inform women on the role of PrEP in HIV prevention was described by one HCP as follows:

“I don't have any cisgender women who want to be on PrEP. I have lots of young men, men who have sex with men, who want to be on PrEP. There's no messaging in the cisgender female population about PrEP. The only people we've captured … they are partners of HIV-positive patients”—DC physician, male

They went on to describe the lack of representation in PrEP marketing as follows:

“Well, all the messaging is not directed at them…it's just not. The public messaging has this…sort of like, you know, it has a…hetero woman there as the token, or they mention something about IVDU [intravenous drug use] and then it… sort of…gets muddied”—DC physician, male

Although most female sex workers in the DR participating in the IDIs were aware of daily oral PrEP, most on daily oral PrEP were generally accessing it as part of small pilot and demonstration projects. Most former or current PrEP users had initially accessed it through a program at a local non-governmental health center providing free PrEP pills. For DR women, word of mouth between friends and sex worker colleagues served as a trusted source of information on HIV and PrEP.

“One must have conversations with the women on different topics until one gains their trust. There are some women who do the same work as me and sometimes we do not trust the information we receive. I trusted my friend because she shared her personal experience about PrEP with me”—DR woman, age 27

In contrast, most DC women did not report talking about HIV or PrEP with their friends or families.

“…it's not even something I've ever had a conversation [on]. Me and my friends talk about a million things. That's one thing I can honestly say we probably never talked about. I just think it's something that a lot of people don't talk about, because a lot of people think like, ‘Oh, well, I'm not getting it,’ or [there are] a lot of misconceptions.…”—DC woman, age 32

Although most participants referred to daily oral PrEP when speaking of PrEP, a subset of participants (particularly HCPs in DC who had been part of prior clinical trials) had heard of LAI PrEP. Knowledge of LAI PrEP among women was very low in both locations, with very few participants reporting any awareness. The HCPs who had heard of LAI PrEP cited social media, published literature, and participation in other HIV prevention studies as sources of information, although most reported not having enough information to confidently speak to patients about it.

### PrEP was often seen as an empowerment tool

Women across sites, including those who had not yet taken PrEP, described PrEP as an empowerment tool that allowed them to exercise control over their HIV risk, thereby putting them in a position of personal power.

“It's like taking control. It's more empowering because you are in control of things…”—DC woman, age 31

Women in both settings described challenges negotiating condom use and lack of control regarding male partner behavior. Women viewed PrEP as a means of protecting themselves from HIV with potentially unfaithful intimate partners. When discussing the perceived value of taking PrEP, one non-PrEP user stated the following:

“I mean it's definitely worth taking, because even if you are in a relationship, you can't always a hundred percent guarantee that they're not going to step out on you”—DC woman, age 32

Participants in the DR reported that clients often offer additional money for sex without a condom and also cited the general HIV risks of sex work such as condoms breaking, non-consensual sex, and regular clients who may feel entitled to condomless sex. In these types of situations, women noted that PrEP gives them a sense of agency in preventing HIV. One current PrEP user noted the following:

“There are men who are bad to you and the condom breaks and you don't know if they broke it on purpose, so it is always good to search and protect yourself [from HIV]. That is why I do not stop taking my pills [daily oral PrEP] and everything is good thank God”—DR woman, age 32

### LAI PrEP may address gender-specific factors and sex work–specific dynamics

Given social-structural factors specific to women, such as the multiple demands of caretaking and balancing work and home life, participants saw added benefits to LAI PrEP.

“It's [LAI PrEP] going to be very convenient because you have to understand, a lot of these women have a lot of competing priorities, right…caregivers, wives, moms, career women, so I think overall it would be a huge convenience”—DC nurse, female

Women in the DR were particularly interested in LAI PrEP given their work dynamics. Female sex workers described how their HIV vulnerability and challenges with daily oral PrEP adherence, including late-night work schedules, drinking as part of work, and the potential for client-related violence, could be addressed by LAI PrEP.

“I arrive home very late because I work in the streets. Sometimes during the day, I have no time, I must take care of my child. I know I won't forget to take a medicine for my own health. But I forget things like that. I told myself that when I took birth control pills, I wouldn't come out pregnant. I got pregnant while taking those pills because sometimes I would forget to take them and take 2 pills the next day”—DR woman, age 30

### PrEP modality options and preferences

Participants in both locations expressed how having more than one option for PrEP would allow women to meet their personal needs. When LAI PrEP was initially described to women, most were interested in learning more about it. One prior PrEP user stated the following:

“I did not know of this other modality for PrEP [LAI PrEP] and I would really like to take it as an injection since taking it every day is easy to forget”—DR woman, age 27

Participants discussed how those with a regular schedule of oral medications may prefer daily oral PrEP because of the ease of incorporating it into their existing routine but that having the injection option could suit others better.

“Most people I know, they take their pills because…I have some friends [who] take prebiotics, like the Azo pills for their urinary tract. I have some friends [who are] like holistic. They do a regimen, so me, I don't have a regimen right now. I have a 6-month-old, so injection is for me”—DC woman, age 31

This sentiment was echoed by a woman in DR:

“I almost take it when I take the pressure pill because when I get up, it's time for me almost, you know because as I work in a business I get up late. So, I do not forget because that is all together there, I cannot forget…they are like a medicine routine”—DR woman, age 43

Women across settings with a fear of needles described being more comfortable with the pill vs. LAI PrEP. Fear of needles was more commonly reported among DC women.

Among women who were concerned about “physical evidence” of using PrEP, LAI PrEP was of particular interest. Participants explained how LAI PrEP could decrease stigma because of the privacy it would afford them; by not requiring them to carry medication, they would not be at risk of having their medication (e.g., pill bottles) discovered by others. Participants cited a lack of knowledge among the general population about PrEP and specifically discussed how HIV treatment medications with the same names as PrEP could lead to individuals falsely assuming one has HIV, whereas LAI PrEP may mitigate potential stigma.

“Sometimes people will see the pills and they ask, ‘What are those pills for?’ They think one is sick and is taking those pills. Like if they have AIDS. They are going to think one takes the pills for AIDS. With the injection, one is not going to have to leave the health center with bottles”—DR woman, age 21

Former or current PrEP users and some HCPs in the DR described a common practice of hiding PrEP pill prescriptions in different medicine bottles or unrelated containers to ensure others would not be able to identify if someone was taking PrEP. When asked which modality would provide greater privacy, an HCP stated the following:

“I understand that the injection would be more confidential because many times the women hide the pill bottles. They can't take it to work, they don't want to be seen with medications and they express it”—DR physician, female

Although women in DC did not generally share similar stories of hiding PrEP pills, women and HCPs in DC echoed the potentially stigmatizing effects of others finding medication bottles.

Some HCPs reinforced the potential of LAI PrEP to reduce stigma among women, particularly those who are most hesitant to adopt PrEP but also are most in need of HIV prevention.

“For folks that feel the stigma of being in need of PrEP, whatever that means to them, whether they interpret that or feel like that can be interpreted as being more promiscuous, or what have you, whatever comes along, whatever they’ve interpreted as being the stigma associated with using PrEP, if an injection allows them—if that's something that's important to them and valuable, and they want to reduce that stigma, an injection would allow that to be reduced. So I think for somebody who's worried about the stigma of using PrEP, I would expect them to be more on board with the injection rather than the pills”—DC physician, female

Healthcare providers were enthusiastic about the arrival of LAI PrEP as another HIV prevention option for women. They liked the idea of clinician-administered LAI PrEP, because then they would know that their clients were protected from HIV for a longer period of time.

“We administer that medication to the user, we already know that she is not going to tell me that she is taking it when she is not, because many times we give the bottles to the patients and they do not take it”—DR physician, female

One DC nurse described the introduction of LAI PrEP as a potential “game-changer” because it gave HCPs and their patients options. This sentiment was echoed by other HCPs who cited LAI PrEP's ability to relieve the pill burden that may turn some potential PrEP users off from initiating use. One DC physician explained that when patients who initially express a general interest in PrEP realize that it must be taken daily, “they lose interest*.*” By having an option that requires less frequent dosing, HCPs were hopeful that PrEP uptake would increase among women.

In both locations, HCPs were also enthusiastic about women in particular being comfortable with and likely adopters of LAI PrEP due to familiarity with other long-acting or injectable birth control medications and felt this familiarity would make it easier for women to relate to receiving the injection on a set schedule.

“…I think it would be more feasible for them because many prefer to use long-acting drugs. That's why when they go to plan they come here for the IMPLANON or for the IUD, so as not to take pills. I think, they will prefer prolonged use of the medication”—DR physician, female

### Supports and services needed to optimize PrEP uptake and use

Across both locations, HCPs highlighted their need for more information about LAI PrEP to be prepared to discuss it with women. Topic areas highlighted included medication administration protocols, a deeper understanding of potential side effects, and dosing protocols. To allow patients the opportunity to truly weigh both PrEP methods and make an informed decision on what would best meet their needs, HCPs wanted to be able to answer any questions their patients may have regarding LAI PrEP.

“Well I think knowing exactly like even small nuts and bolts like how big the needle is, how much fluid gets injected, how long the clinic visit would be every 2 months, is there anything we're going to be asking them to do other than get this injection at these visits, do they need blood pressure and vital signs, how often since, we have somebody on PrEP we think they're in moderate to higher risk of HIV, how often–do they have to do an HIV test every time? How often are we going to do other STD test offerings. Those sorts of things are going to help in providing a realistic expectation of what this every-2-month thing is going to look like”—DC nurse, male

Financial support was a pressing concern for HCPs and women. In the DR, HCPs perceived the cost of LAI PrEP as a barrier to women using it and expressed interest in financial incentives for sustaining LAI PrEP use. They cited that daily oral PrEP was currently free and that LAI PrEP would also need to be free for women to adopt this modality.

“They [female sex workers] generally go for the method that is free, so for me, it is important it remains free”—DR health administrator, female

However, women in the DR expressed that the benefits of any modality of PrEP overcame any high cost associated with its use.

“I don't care much about the price, I don't care if it is RD$3000 ($55 USD), RD$4000 ($74 USD). If I don't have enough, I will ask them to sell it to me and I will pay it in parts, understand? And if I don't buy it in parts and don't have the money, I will ask for a loan”—DR woman, age 43

In DC, HCPs and women highlighted the need for insurance to mitigate the cost of LAI PrEP to hasten uptake. Healthcare providers were interested in understanding if LAI PrEP would be covered in the same way that daily oral PrEP is covered by federal health programs. Women wanted to know up front how much their personal monetary cost (co-pay) would be so that they could be prepared to pay the expense.

“Because myself, I'm not just rolling around in finances, so every penny is accounted for daily. So it gets spread around for all types of bills, and adding an extra bill would have to be something that's added into finances, to be able to budget. Once again, I think something like this should be, help fund it in some way”—DC woman, age 36

Other logistical support highlighted by women and HCPs included transportation, appointment reminders, and childcare. Women in DC cited clinic policies that did not allow children under the age of 18 in the office, which further exacerbated their need for childcare assistance. Some proposed solutions included having special clinic hours on evenings or weekends that would allow more flexibility with finding childcare.

“We also must know that arriving to the visit costs money and arriving to the visit at a time that conflicts with work hours is also costly because you either lose one day of work or need to request time off, so [addressing] those elements that you see as a lot of work will really facilitate [uptake]”—DR provider, male

Healthcare providers also highlighted the potential for using pharmacies with attached clinics as locations in the community that may address potential concerns around privacy and appropriate expertise for administering LAI PrEP. They also discussed the potential of stigmatization based on the type of clinic that provides LAI PrEP, citing the added anonymity of attending a large medical center that provides multiple services under one roof and privacy of care for those who attend.

“When you go to the doctor's office you just say, ‘Oh, I need to get a shot.’ It's pretty common that people go for something or that you just have to go in for a follow-up visit. You don't necessarily need to say that you're going in for an injection. You're just going in”—DC physician, female

### Quantitative findings

#### Sociodemographic characteristics of survey participants

As shown in Table 2, women in the quantitative sample (DC, *n* = 302; DR, *n* = 300) had a mean (SD) age of 31 (8.8) and 33 (11.0) years and 1.7 (1.7) and 2.5 (2.4) children in DC and the DR, respectively. Most were single (DC, 64%; DR, 80%). Among DC participants, 86% were Black/African American; 53% had a high school education and 23% had some college; 36% were unemployed, whereas 41% reported full-time employment; 86% were on Medicaid or Medicare, whereas 17% had private health insurance; 11% reported being unstably housed; and most (77%) were surveyed at a family planning and preventive care clinic, whereas 23% were surveyed at a general obstetrics and gynecology clinic. Among DR participants, 55% had completed secondary school; 84% reported fair or poor financial security; and all reported sex work as one of their forms of livelihood.

**Table 2:** Characteristics of quantitative sample.

Characteristic, *n* (%)^a^	Women (*N* = 602)	
	DC (*n* = 302)	DR (*n* = 300)
Sociodemographic		
Age, mean (SD), years	31 (8.8)	33 (11.0)
Current relationship status		
Single/Never married	192 (64)	241 (80)
In a steady relationship, but not living together	26 (9)	–
In a steady relationship and living with my partner(s)	36 (12)	40 (13)
Legally married^b^	33 (11)	7 (2)
Divorced	4 (1)	0
Separated	7 (2)	6 (2)
Widowed	4 (1)	3 (1)
Prefer not to answer	–	3 (1)
Number of children, mean (SD)^c^	1.7 (1.7)	2.5 (2.4)
Health		
Average number of pills each day for health issues, including any vitamins and supplements		
0	142 (47)	189 (63)
1	65 (22)	64 (21)
2–3	71 (24)	41 (14)
4–5	22 (7)	5 (2)
≥6	2 (1)	1 (<1)
Have you ever used injectable medications (including injectable contraception)?		
No	158 (52)	166 (55)
Yes	144 (48)	134 (45)
Have you ever used any recreational (street) drugs by injection?		
No	287 (95)	298 (99)
Yes	15 (5)	2 (1)
STI test (past 6 months)		
No	101 (33)	184 (61)
Yes	201 (67)	116 (39)
Diagnosed with an STI (past 6 months)		
No	269 (89)	291 (97)
Yes	33 (11)	9 (3)
What do you think the chances are that you will ever get HIV?		
Very likely	9 (3)	65 (22)
Somewhat likely	28 (9)	75 (25)
Unlikely	212 (70)	124 (41)
Not very likely	53 (18)	36 (12)
Ever HIV test		
No	44 (15)	17 (6)
Yes	258 (85)	283 (94)
Any sexual partners physically harmed you (past 6 months)^d^		
No	260 (94)	263 (88)
Yes	17 (6)	37 (12)
Substance use		
How often do you have a drink containing alcohol?		
Never	90 (30)	15 (5)
Monthly or less	89 (30)	47 (16)
2–4 times a month	71 (24)	69 (23)
2–3 times a week	36 (12)	93 (31)
4 or more times a week	16 (5)	76 (25)
How many drinks containing alcohol do you have on a typical day when you are drinking?^e^		
1–2	148 (70)	25 (9)
3–4	47 (22)	44 (15)
5–6	10 (5)	39 (14)
7–9	5 (2)	35 (12)
≥10	2 (1)	142 (50)
How often do you have ≥6 drinks on one occasion?^e^		
Never	110 (52)	23 (8)
Less than monthly	66 (31)	21 (7)
Monthly	29 (14)	48 (17)
Weekly	6 (3)	150 (53)
Daily or almost daily	1 (<1)	43 (15)
Have you ever used any recreational drugs in your lifetime?		
No	197 (65)	222 (74)
Yes	105 (35)	78 (26)
Drug used in last 6 months (check all that apply)		
Marijuana	93 (31)	54 (18)
Cocaine	9 (3)	15 (5)
Heroin	2 (1)	3 (1)
Tobacco	19 (6)	146 (49)
Ecstasy	6 (2)	4 (1)
Crystal meth	2 (1)	–
Prescription drugs, not as intended	2 (1)	–
Prescription drugs that were not prescribed to me	2 (1)	–
None	8 (3)	–
Other substance(s), (specify)	10 (3)	4 (1)

DC, Washington, District of Columbia; DR, Dominican Republic; STI, sexually transmitted infection.

a Unless otherwise noted.

b DR participants response option was “married with papers or living together”.

c DC participants were asked how many children they have, whereas DR participants were asked how many biological children they have living.

d DC population denominator, *n* = 277.

e DC population denominator, *n* = 212; DR population denominator, *n* = 285.

### Low perceived HIV risk and indications for PrEP use

HIV risk perception among both populations was relatively low but particularly so among women in DC. An overwhelming 88% of participants in DC reported it was unlikely/not very likely they will ever get HIV, compared with 53% of participants in the DR (Table 2). Despite low perceived risk, two-thirds (67%) of women in DC reported having tested for an STI in the past 6 months, with 11% receiving an STI diagnosis; in the DR, 39% reported having an STI test and 3% reported receiving an STI diagnosis in the past 6 months (Table 2). Due to local health system burden and quality of care challenges among female sex workers in the DR (28), the 3% reported rate for STI diagnoses may be artificially low due to uncommunicated diagnoses.

Sixteen percent of DC participants reported 2 or more main sexual partners (defined as someone you feel committed to above anyone else); 18% reported 2 or more causal partners (defined as someone you do not feel committed to); 5% exchanged sex for money, drugs, food, shelter, or other reason. More than half (57%) of DC participants reported never using condoms in the past 6 months, and 68% had an HIV test in the past year. Of DC participants who knew of PrEP, nearly 15% reported never having used PrEP because they did not feel they had reasons to use HIV prevention. Among female sex workers in the DR, 79% reported 3 or more sex acts in the past week, with 65% reporting consistent condom use (always using condoms) with new clients, 81% reporting consistent condom use with regular clients, and 32% reporting consistent condom use with non-client partners; 59% had tested for HIV in the past 6 months. When asked if any sexual partners physically harmed participants in the past 6 months, 6% and 12% of DC and DR participants, respectively, reported yes (Table 2).

In terms of substance use, 17% of participants in DC reported drinking alcohol 2 or more times weekly, compared with 56% in the DR, and 35% of DC participants reported ever using drugs, compared with 26% of DR participants (Table 2).

### Very limited awareness of PrEP in both settings

Data revealed very limited PrEP awareness across both sites, with 71% of DC participants and 94% of DR participants reporting never having heard of daily oral PrEP. Moreover, higher percentages of participants in both sites were unaware of LAI PrEP (DC, 91%; DR, 98%). Only 3% and 1% of DC and DR participants, respectively, had ever used PrEP. When asked why they had never used PrEP, 62% of DC participants selected that they had never heard of it.

Among the small percentage of DC participants aware of daily oral PrEP, 50% had heard of it from advertisements or social media, 30% from an HCP, and 24% from a friend or family member. In the DR, those aware of daily oral PrEP had heard about it from a friend or family member (41%), an HCP (29%), or a sexual partner or sex worker (24%). More than one-third (36%) of participants in DC and more than three-quarters (78%) in the DR did not know how to start the process to obtain PrEP if they wanted to use it, but 48% in DC and 73% in the DR said they would feel comfortable talking to an HCP at the clinic where they were being surveyed about initiating PrEP. Most participants said they would find it useful to have a peer navigator supporting them in PrEP uptake and use (DC, 66%; DR, 97%).

### Sites varied in level of interest in PrEP but general preference was for LAI PrEP

Levels of interest in PrEP highly varied across sites, with only 5% of DC participants reporting definite interest in using some form of PrEP, compared with 80% of DR participants. Variability was also observed among those reporting definitely no interest in some form of PrEP (DC, 30%; DR, 3%). However, both sites had similar levels of preference for LAI vs. daily oral PrEP, with 73% in DC and 71% in the DR indicating a preference for LAI PrEP every 2 months vs. daily oral PrEP (Figure 1). In terms of locations where they would want to obtain PrEP, DC participants preferred family planning clinics (64%), primary care clinics (59%), sexual health clinics (38%), and pharmacies (29%); DR participants preferred pharmacies (32%), sexual health clinics (31%), and community centers/organizations (21%).

**Figure 1:**
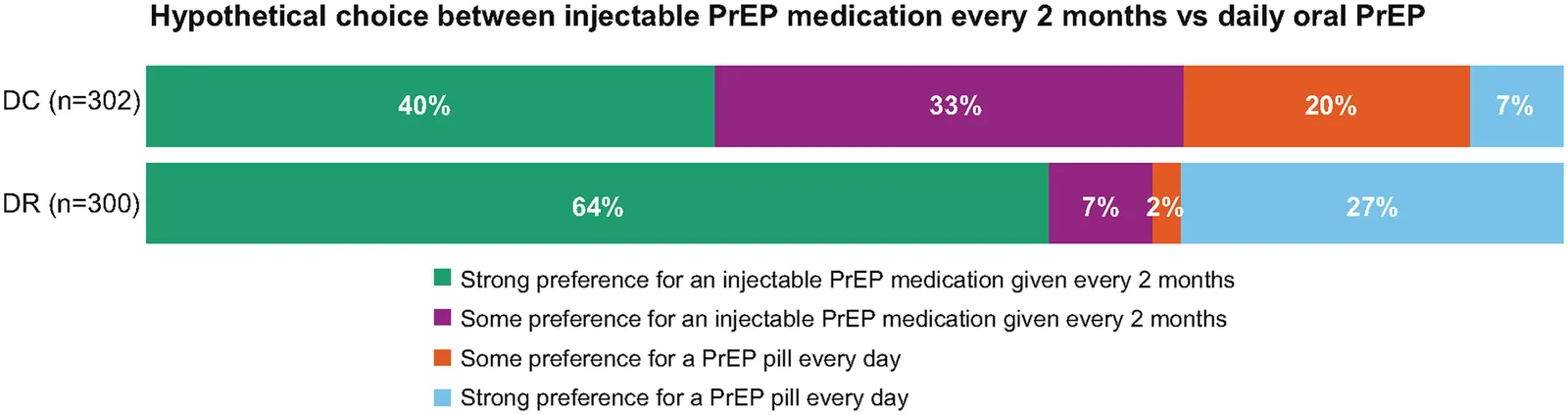
PrEP modality preferences among participants.

Across sites, proportions reporting previous injection experience with medications or contraception (DC, 48%; DR, 45%) or taking ≥1 pill per day (DC, 53%; DR, 37%) were generally similar. In terms of general medication preferences, comparable proportions reported that they would prefer an injection to swallowing a pill (DC, 49%; DR, 70%), that it would be easier for them to go to a clinic every 2 months than remembering to take a pill every day (DC, 65%; DR, 60%), and that they would be more worried about having others discover PrEP pills than being seen at a PrEP clinic (DC, 60%; DR, 56%).

### PrEP modality considerations

Regarding PrEP side effects, abdominal distress was of greatest concern among both sites, with 73% and 62% of DC and DR participants indicating that they would be bothered about abdominal distress. Injection site pain was also reported as a concerning side effect (DC, 54%; DR, 49%). Weight gain was the next most concerning side effect among both sites, with 47% in DC and 39% in DR indicating that they would be bothered about 1–2 pounds of weight gain over the course of a year from either form of PrEP modality (bothered about ≥4 pounds of weight gain: DC, 56%; DR, 43%). Finally, 36% of DC participants and 22% of DR participants reported that they would be very concerned about the cost of PrEP medication (including co-payments and out-of-pocket costs).

### Shared and unique factors associated with LAI PrEP preference across sites

In bivariate analysis among DC participants, preference for LAI PrEP was associated with reporting having had an HIV test at any time (OR, 1.91 [95% CI, 0.98–3.73]; *P* = 0.06), using recreational drugs at any time (OR, 1.75 [95% CI, 0.99–3.09]; *P* = 0.05), not being very bothered by a potential side effect of 1–2 pounds of weight gain over the course of a year (OR, 1.62 [95% CI, 0.77–3.42]; *P* = 0.05), and a higher score on the PrEP optimization scale (OR, 1.06 [95% CI, 1.01–1.10]; *P* = 0.01). Preference for LAI PrEP was negatively associated with reporting extreme injection fear (OR, 0.31 [95% CI, 0.15–0.65]; *P* < 0.01), extreme fear of needles (OR, 0.32 [95% CI, 0.16–0.67]; *P* = 0.03), and being very bothered by injection site pain or tenderness (OR, 0.54 [95% CI, 0.25–1.18]; *P* = 0.03). Higher scores on the PrEP stigma scale were negatively associated with LAI PrEP preference.

In bivariate analysis among DR participants, preference for LAI PrEP was associated with reporting prior injection experience with medications/contraception (OR, 2.41 [95% CI, 1.42–4.11]; *P* < 0.01), being bothered by weight gain of 1–2 pounds (OR, 1.98 [95% CI, 1.04–3.76]; *P* = 0.04), a higher score (19–21) on the PrEP optimization scale (OR, 2.10 [95% CI, 1.06–4.18]; *P* = 0.03), and higher (≥7 drinks per day per month) monthly alcohol use (OR, 1.85 [95% CI, 0.98–3.49]; *P* = 0.06). Preference for LAI PrEP was negatively associated with reporting being afraid of injections (OR, 0.09 [95% CI, 0.04–0.20]; *P* < 0.01), experiencing partner violence (OR, 0.43 [95% CI, 0.21–0.86]; *P* < 0.02), and a higher score on the PrEP stigma scale (OR, 0.92 [95% CI, 0.87–0.97]; *P* < 0.01).

Prior to running the multivariate analyses per site, we also explored the effect of site on the primary outcome of LAI preference by running a simple logistic regression with site (DR as the reference site) as the sole predictor variable. In this exploration, we found no significant differences in the odds of preferring LAI PrEP across sites (OR 1.11; 95% 0.78–1.59; *P* = .55).

Among DC participants, the final multivariate model (Table 3) revealed LAI PrEP preference was significantly positively associated (at *P* < 0.05 level) with a higher score on the PrEP optimization scale (aOR, 1.06 [95% CI, 1.01–1.10]; *P* = 0.01) and significantly negatively associated with reporting being bothered by injection site pain or tenderness as a side effect (aOR, 0.54 [95% CI, 0.31–0.96]; *P* = 0.04).

**Table 3:** Logistic regression models of preference for LAI PrEP: DC.

Variable	Full model			Final model		
	Adjusted OR	95% Cl	*P* value	Adjusted OR	95% Cl	*P* value
Age (years)	1.00	0.97–1.04	0.72	–	–	–
Ever HIV test	1.88	0.93–3.79	0.08	1.90	0.95–3.80	0.07
Side effect: injection site pain or tenderness	0.55	0.31–0.97	0.04	0.54	0.31–0.96	0.04
PrEP optimization	1.06	1.01–1.10	0.01	1.06	1.01–1.10	0.01
PrEP stigma	0.96	0.92–1.01	0.09	0.96	0.92–1.00	0.09

DC, Washington, District of Columbia; LAI, long-acting injectable; PrEP, pre-exposure prophylaxis.

Among DR participants, the final multivariate model (Table 4) revealed that reporting prior injectable medicine or injectable contraceptive experience (aOR, 2.39 [95% CI, 1.37–4.18]; *P* < 0.01) and higher alcohol use (aOR, 1.90 [95% CI, 1.11–3.25]; *P* = 0.02) were significantly associated with LAI PrEP preference. Being bothered by injection site pain or tenderness as a side effect was significantly negatively associated with LAI PrEP preference (aOR, 0.46 [95% CI, 0.23–0.93]; *P* = 0.03), as was sexual partner violence (aOR, 0.39 [95% CI, 0.18–0.82]; *P* = 0.01) and a higher score on the PrEP stigma scale (aOR, 0.93 [95% CI, 0.88–0.99]; *P* = 0.03).

**Table 4:** Logistic regression models of preference for LAI PrEP: DR.

Variable	Full model			Final model		
	Adjusted OR	95% Cl	*P* value	Adjusted OR	95% Cl	*P* value
Age (years)	1.00	0.97–1.02	0.74	–	–	–
Income (per 1,000 Dominican pesos)	1.00	0.99–1.02	0.26	1.01	0.99–1.02	0.20
Average no. of pills per day >0	0.73	0.42–1.28	0.27	–	–	–
Injection experience (injectable medicine and/or contraceptives)	2.41	1.35–4.30	<0.01	2.39	1.37–4.18	<0.01
Side effect: injection site pain or tenderness	0.47	0.23–0.96	0.04	0.46	0.23–0.93	0.03
Alcohol use						
>Monthly and <7 drinks/day	0.85	0.40–1.82	0.68	–	–	–
>Monthly and ≥7 drinks/day	1.59	0.79–3.17	0.19	1.90	1.11–3.25	0.02
Sexual partner violence	0.45	0.20–0.98	0.05	0.39	0.18–0.82	0.01
PrEP optimization	1.08	0.94–1.25	0.27	–	–	–
PrEP stigma	0.94	0.88–1.00	0.05	0.93	0.88–0.99	0.03
Sex worker stigma: shame	0.97	0.91–1.04	0.39	–	–	–

DR, Dominican Republic; LAI, long-acting injectable; PrEP, pre-exposure prophylaxis.

## Discussion

This mixed methods study provides unique sights into awareness, interest and uptake of daily oral and LAI PrEP among women who could benefit from PrEP. By examining the perspectives and experiences of women from differing backgrounds, and their providers, across settings study findings offer insights into how programming can be shaped to address the needs of diverse groups of women. In the case of women recruited in Washington DC, findings reflect the experiences of Black, generally lower-income women, while in the DR results reflect the experiences of female sex workers. In both cases these groups are faced with intersectional stigma and discrimination related to gender, race, and class shaping their experiences with the healthcare system and their relationships (16, 29). In the following section we discuss findings and intervention implications for introducing and sustaining access and adherence to PrEP.

### Low awareness of PrEP and especially LAI PrEP across settings

Both qualitative and quantitative data collection conducted through this research underscore previously documented low awareness of PrEP overall among women in domestic and international settings (30–33) and among women who may particularly benefit from PrEP (10, 31, 34, 35), including female sex workers. Both IDIs and survey data also showed very limited awareness of LAI PrEP. Only 9% of surveyed study participants in DC had heard of LAI PrEP despite its having received FDA approval in December 2021 (survey data collection, November 2021 to February 2022). Healthcare providers participating in the qualitative aim of this study observed that they would need to know more about LAI PrEP before feeling comfortable presenting it as an option for their patients, further limiting patient awareness of LAI PrEP. Both qualitative and quantitative findings indicate the critical importance of further PrEP awareness campaigns that provide up-to-date and accurate information focused on the needs and priorities of women, and trainings for their providers particularly regarding LAI PrEP.

Study participants in both our qualitative and quantitative research who had heard of PrEP typically felt that PrEP was for other populations (e.g., men who have sex with men) and not for them. In DC, HCPs participating in our qualitative research also noted the lack of PrEP messaging specific to women. These findings mirror research showing that women in the US experience a lack of PrEP marketing tailored to them (10, 29, 36–38). Across settings, research has shown that a lack of marketing and information on PrEP presents a barrier to PrEP uptake (29, 36, 39, 40).

### Importance of HCPs in PrEP awareness and decision-making

Our quantitative research also revealed the potential role of HCPs in contributing to PrEP awareness. In DC and DR, nearly one-third of participants had heard of daily oral PrEP from their HCPs. Previous research has also shown that HCPs are an important source of PrEP awareness among women in DC (41) and female sex workers in global settings (38). In addition to ensuring that women are aware that PrEP is an option for them, our research suggests that women may be open to discussing with their HCP whether and which kind of PrEP is right for them. Nearly half (48%) of surveyed participants in DC and most (73%) in the DR said they would feel comfortable talking to an HCP about PrEP at the clinic where they were being surveyed for this research. Other research in the US has shown that most patients are comfortable discussing PrEP with their HCPs (42) and want their HCP to discuss PrEP with them in sexual and reproductive health visits (37, 43). Research has also shown that women who are comfortable discussing PrEP with their HCP have a significantly greater likelihood of using PrEP (44).

Most surveyed DC participants would be willing to obtain PrEP in a family planning (64%) or primary care clinic (59%), whereas one-third (31%) of DR participants expressed a willingness to obtain PrEP in a sexual health clinic. These results suggest reproductive health and primary care clinics offer an acceptable platform for increasing awareness of PrEP and PrEP options for women. The high numbers of participants in both settings who reported having received recent HIV and STI testing also suggest that they are engaged in healthcare and that this ongoing engagement presents an opportunity to discuss the availability of PrEP and PrEP modality options.

### Elevated likelihood of acquiring HIV and low perceived reasons for HIV prevention

Although some women in both settings self-reported recent behavioral or health histories indicating a potential benefit of initiating PrEP, most perceived their likelihood of acquiring HIV to be low. Eleven percent of surveyed participants in DC reported an STI diagnosis in the last 6 months. In DC, prevalence of HIV at both a population level and among Black women is 2%, and the CDC has designated DC as an “HIV hotspot” (41, 45). However, most surveyed DC participants (88%) reported that it is unlikely or not very likely they will acquire HIV. In the DR, the national prevalence of HIV is 5% among sex workers (46). One-third of surveyed DR participants do not consistently use condoms with new clients and two-thirds do not consistently use condoms with non-client partners. Despite this, 53% of DR women reported that it is unlikely or not very likely that they would acquire HIV. Having a low perception of likelihood of HIV acquisition while actually being in a situation that increases that likelihood (social determinants and location) is common in US (31, 47, 48) and female sex worker populations (49), while concerns about stigma and discrimination have been shown to limit women's comfort and willingness to disclose their potential reasons for HIV prevention to HCPs (42, 50). Findings from this research indicate a lower than actual perceived need for HIV prevention among women, indicating the importance of HCPs uniformly entering into discussions with their female patients about the availability of PrEP and PrEP choices, where applicable, regardless of whether PrEP interest and indications are disclosed. These findings indicate the importance of promoting PrEP conversations between women and providers that are not based on self-reported risk so not to exclude women who could benefit from PrEP but may not disclose risk.

### PrEP seen as an empowerment tool for women

A key qualitative finding from this study was around the potential for PrEP to serve as an empowerment tool for women. In contemplating PrEP options, women in this study revealed layers of social-structural constraints, including gender-related norms, pressures, and responsibilities, as well as inequitable partner dynamics and partner violence, that lie as the backdrop to their PrEP decision-making. These contextual factors have also been documented in prior studies citing the role of social-structural factors, such as childcare responsibilities (51), financial insecurity (51, 52), and structural violence (36), which affect women's perspectives and interest in PrEP. Given complex structural vulnerabilities coupled with individual risk factors, PrEP was seen by participants in this study as an HIV prevention option that would allow them greater autonomy. Prior studies have documented the importance of agency and potential empowerment provided by PrEP as an HIV prevention method (53).

### Peer and community support can optimize PrEP uptake and decision-making

Qualitative findings from this study highlight the importance of peer and community support in optimizing PrEP uptake and decision-making among women. Although DC women were less familiar with peer navigators than DR women, the idea of hearing from other women who had used PrEP to learn from their experiences clearly resonated with them and was reflected in quantitative findings. Two recent studies with Black women in the US echo this finding, with participants identifying that barriers to PrEP could be addressed by partnering with PrEP-engaged Black women (e.g., for testimonials) (29), as well as support groups, community-level programs, and education (35). In the context of sub-Saharan African populations, findings from a PrEP demonstration project in Tanzania and South Africa found that adherence clubs among peers were a helpful social support strategy, particularly in gender-inequitable sexual relationships (54). In a prior study of female sex workers in Tanzania, participants also described community-led peer education as key to delivering trusted information on PrEP to female sex worker communities (16). Current study findings contribute to this literature supporting the importance of peer involvement and support in PrEP uptake and use among women.

### PrEP modality preferences and factors driving preferences among women

In both settings, women preferred LAI over oral PrEP. These findings mirror other studies with women in the literature.

In a recent study among US women, 55% of participants reported that they would prefer LAI PrEP (compared with 10% preferring daily oral PrEP), citing ease of use, convenience, confidentiality, and perceived greater effectiveness vs. daily oral PrEP (55). Our prior research among female sex workers in Tanzania found that 88% preferred LAI over daily oral PrEP, noting its convenience, confidentiality, and perceived greater effectiveness vs. daily oral PrEP (10). Moreover, among female sex workers, LAI PrEP has been described as an attractive option given often unpredictable work schedules; sex work−related substance use, such as alcohol; and elimination of the need to carry or be seen with pills that can be linked to HIV and potentially increase experiences of stigma, discrimination, or violence (15).

Qualitative and quantitative findings from the current study also offer important insights into factors influencing preference for PrEP modality among women. Among DC participants, a higher score on the PrEP optimization scale was significantly associated with preference for LAI PrEP, suggesting that having the opportunity to consider and weigh options is important to women in the US so they can optimize PrEP care. Although this optimization process did not emerge as a factor significantly associated with LAI PrEP preference in multivariable analysis in DR, it is possible that this did not resonate as much among DR participants given that LAI PrEP is not yet an option in DR. Prior qualitative work with female sex workers in Tanzania supports the importance of women understanding both options to ensure informed decision-making around PrEP modality and underscores that women want to weigh their options to help them determine whether they are better suited for daily oral or LAI PrEP (16).

DR female sex workers' preference for LAI over daily oral PrEP echoes a recent systematic review indicating female sex workers' preference for LAI PrEP globally (38). Alcohol use emerged in our data as a female sex worker–specific factor in predicting LAI PrEP preference in the DR. Prior research suggests female sex worker–specific factors that alleviate occupational barriers to daily oral PrEP, including alcohol use and unpredictable work schedules, contribute to LAI PrEP preference among female sex workers (16). Given daily occupational realities, LAI PrEP was seen by female sex workers as having the potential to reduce stress related to daily oral PrEP (16).

Among both DC and DR participants, concern about potential side effects, specifically injection site pain, was a significant factor in PrEP preference. Concerns about potential side effects of PrEP among women are well documented in the literature (29, 35, 36), particularly concern around injection site pain as a barrier to LAI PrEP uptake (20). However, a history of injectable medicines or contraceptives was significant in quantitative findings among DR participants; moreover, qualitative findings among both DC and DR participants highlight that women are connecting their experiences and familiarity with long-acting contraception to the acceptability of LAI PrEP. A recent study showed direct associations between women being more interested in injectable PrEP if they had used injectable contraceptives and more interest in daily oral PrEP if they had used oral contraceptives (56), highlighting a natural entry point for conversations connecting contraceptive use with selection of PrEP modality.

Findings reveal that PrEP stigma was salient for DR female sex workers and though present, was less pervasive among DC participants. Previous research documented that PrEP stigma plays a role in PrEP decision-making (31, 36, 42, 52). It is also important to consider an intersectional stigma lens for understanding PrEP preferences among the women in this study. Both Black women living in DC and female sex workers in the DR are historically marginalized groups who may experience stigma as women, as people of color, and due to socioeconomic status, among other reasons. As evidenced from this study's findings as well as other studies with Black women (57), PrEP is commonly associated with men who have sex with men and transgender individuals; thus, using PrEP has the potential to introduce additional layers of stigma to an already stigmatized group. Here we observed that greater PrEP stigma and sexual partner violence was significantly negatively associated with LAI PrEP preference among DR participants. Further research to unpack these dynamics is needed to help understand how PrEP preference is affected by stigma and sexual violence; for instance, do increased clinic visits for LAI PrEP outweigh the potential benefit of not having to carry pills. Qualitative findings from this study as well as prior research (28) highlight the need for women-centered marketing and engagement strategies to address stigma and partner violence and support PrEP uptake among women.

### Key supports and services needed for PrEP uptake and decision-making

Finally, the current study underscores the need for key supports and services to optimize PrEP uptake and decision-making. Current findings highlight the importance of financial and logistical support (e.g., transportation, childcare) to facilitate PrEP uptake and use among women as has been previously documented (58), as well as insurance assistance for women in DC and cost coverage for women in the DR. In terms of tools to support shared decision-making around PrEP, HCPs in the DR expressed the need for HCP trainings and patient-provider shared decision-making aids to support conversations around PrEP modality selection with patients, highlighting the need for materials outlining advantages and disadvantages of each PrEP modality. Moreover, HCPs felt that materials that could be sent home with potential PrEP users to continue considering key points from the conversation with their HCP would be helpful for the decision-making process for women-first learning of PrEP and wanting to consider and weigh options. Future research should consider the introduction of communication and shared decision-making tools in women's health clinics including reproductive, family planning, and obstetrics and gynecology clinics where HCPs can introduce PrEP into conversations around sexual health using a non-judgmental approach which normalizes PrEP conversations.

### Limitations and strengths

This study is not without its limitations, including the cross-sectional nature of both the qualitative and quantitative data, as well as reliance on self-reported data. Additionally, the study was conducted around the same time as FDA approval of LAI PrEP; potentially limiting awareness and training around LAI PrEP. The study was not designed to be generalizable to all women in both geographic contexts. Despite these limitations, the mixed-methods approach and triangulation of data sources are important strengths of the study. Additionally, lessons learned from findings may be transferrable to similar groups of women in the US or female sex workers in low- and middle-income countries and can inform next research steps as well as tailored multi-level interventions to support daily oral and LAI PrEP uptake.

## Conclusions

Although PrEP awareness, especially LAI PrEP awareness, was low among participating women from Washington, DC and the Dominican Republic, most would welcome having a choice in PrEP modality-underscoring the critical importance of expanding access to PrEP options. Participating HCPs often lacked the confidence and support to engage in discussions around PrEP, especially LAI PrEP, despite it being a preferred option for many women. Supports and services are needed to address social-structural factors among women to reduce disparities in PrEP uptake, including efforts to ensure equitable access to both daily oral and LAI PrEP in resource-constrained settings. These multi-level interventions should be tailored to the needs of specific subgroups of women and include women-centered awareness campaigns and HCP trainings and tools that promote shared decision-making not predicated on self-reported risk assessments.

## Data Availability

The datasets presented in this article are not readily available because data cannot be shared publicly because of potentially sensitive information from a vulnerable population. De-identified data requests from researchers who meet the criteria for access to confidential data should be directed to the first author. Study instruments including scale items are available upon requested to the corresponding author. Requests to access the datasets should be directed to Deanna Kerrigan, deanna.kerrigan@rutgers.edu.
